# Fermentable soluble fibres spare amino acids in healthy dogs fed a low-protein diet

**DOI:** 10.1186/s12917-016-0752-2

**Published:** 2016-06-28

**Authors:** Wendy Wambacq, Galena Rybachuk, Isabelle Jeusette, Kristel Rochus, Brigitte Wuyts, Veerle Fievez, Patrick Nguyen, Myriam Hesta

**Affiliations:** Laboratory of Animal Nutrition, Department of Nutrition, Genetics and Ethology, Faculty of Veterinary Medicine, Ghent University, Heidestraat 19, B-9820 Merelbeke, Belgium; Department of Research and Development, Affinity Petcare SA, Sant Cugat Nord Office Park, Xavier Cugat Square, Building D, 08174 Sant Cugat del Valles, Barcelona Spain; Laboratory of Metabolic Disorders, Department of Clinical Chemistry, Microbiology and Immunology; Ghent University Hospital, De Pintelaan 185, B-9000 Ghent, Belgium; Laboratory for Animal Nutrition and Animal Product Quality, Department of Animal Production, Faculty of Bioscience Engineering, Ghent University, Proefhoevestraat 10, 9090 Melle, Belgium; Nutrition and Endocrinology Unit, Nantes-Atlantic National College of Veterinary Medicine, UNAM Université, Food Science and Engineering-ONIRIS, Site de la Chantrerie, BP 40706, 44307 Nantes Cedex 3, France

**Keywords:** Acylcarnitine, Apparent protein digestibility, Canine renal diet, Dietary fibre, Guar gum

## Abstract

**Background:**

Research in cats has shown that increased fermentation-derived propionic acid and its metabolites can be used as alternative substrates for gluconeogenesis, thus sparing amino acids for other purposes. This amino acid sparing effect could be of particular interest in patients with kidney or liver disease, where this could reduce the kidneys’/liver’s burden of N-waste removal. Since dogs are known to have a different metabolism than the obligatory carnivorous cat, the main objective of this study was to assess the possibility of altering amino acid metabolism through intestinal fermentation in healthy dogs. This was studied by supplementing a low-protein diet with fermentable fibres, hereby providing an initial model for future studies in dogs suffering from renal/liver disease.

**Results:**

Eight healthy dogs were randomly assigned to one of two treatment groups: sugar beet pulp and guar gum mix (SF: soluble fibre, estimated to mainly stimulate propionic acid production) or cellulose (IF: insoluble fibre). Treatments were incorporated into a low-protein (17 %) extruded dry diet in amounts to obtain similar total dietary fibre (TDF) contents for both diets (9.4 % and 8.2 % for the SF and IF diet, respectively) and were tested in a 4-week crossover feeding trial. Apparent faecal nitrogen digestibility and post-prandial fermentation metabolites in faeces and plasma were evaluated. Dogs fed the SF diet showed significantly higher faecal excretion of acetic and propionic acid, resulting in a higher total SCFA excretion compared to IF. SF affected the three to six-hour postprandial plasma acylcarnitine profile by significantly increasing AUC of acetyl-, propionyl-, butyryl- + isobutyryl-, 3-OH-butyryl-, 3-OH-isovaleryl- and malonyl-L-carnitine. Moreover, the amino acid plasma profile at that time was modified as leucine + isoleucine concentrations were significantly increased by SF, and a similar trend for phenylalanine and tyrosine’s AUC was found.

**Conclusion:**

These results indicate that guar gum and sugar beet pulp supplementation diminishes postprandial use of amino acids favoring instead the use of short-chain fatty acids as substrate for the tricarboxylic acid (TCA) cycle. Further research is warranted to investigate the amino acid sparing effect of fermentable fibres in dogs with kidney/liver disease.

## Background

Intestinal microbial fermentation of fibre is considered to be beneficial for host health [1, 2]. Guar gum, a fibre source that is not digestible by host enzymes [3], is known to specifically stimulate the production of fermentation-derived propionic acid in cats [4, 5]. Previous research in this species has shown that increased fermentation-derived propionic acid and its metabolites can be used as alternative substrates for gluconeogenesis, thus sparing amino acids for other purposes (the AA-sparing effect) [6, 7]. However, guar gum may have a negative effect on faecal consistency [8], and is best combined with beet pulp, known for its positive effect on faecal consistency [9]. Additionally, fermentable soluble fibres may alter the host N-metabolism. Increased intestinal luminal water drawn by viscous dietary fibre (the solvent drag effect) and increased bacterial population (due to increased energy supply for fermentation) will contribute to N removal from the blood, incorporating this N in bacterial protein that is excreted through the faeces, resulting in a nitrogen-trap effect (N-trap) [10, 11]. This N-trap and amino acid (AA)-sparing effect could be of particular interest in patients with kidney or liver disease, where the usage of guar gum in combination with beet pulp could allow for a decrease in the protein content of the diet without risking protein malnutrition and, at the same time reduce the kidneys’ and/or liver’s burden of N-waste removal.

The present study aimed to evaluate whether microbial fermentation of soluble fibre in healthy adult dogs could increase production of propionic acid in the large intestine, resulting in increased concentrations in faeces and its metabolites (acylcarnitines) in plasma. It was hypothesized that fermentable fibre supplementation of a low-protein (renal type) diet, compared to insoluble fibre supplementation, will lead to amino acid (AA)-sparing and nitrogen (N)-trap in this species. To investigate this effect, N-balance, plasma acylcarnitine profile and selected parameters of protein metabolism (serum biochemistry and plasma AA concentrations as well as body composition) were evaluated in this study.

## Methods

### Experimental design

Two experimental, low protein, renal-type diets containing either soluble (SF) or insoluble fibre (IF) were tested in a blind, cross-over design. The study consisted of two consecutive periods of 4 weeks. Each period entailed an adaptation period of 23 days to allow the animals to adapt to one of the two diets and 5 days of sample collection. The research protocol was evaluated and approved by the Ethical Committee of the Faculty of Veterinary Medicine, Ghent University, Belgium (EC 2013/74) and was in accordance with institutional and national guidelines for the care and use of animals.

### Animals & housing

A total of eight healthy adult Beagle dogs, bred for research purposes (Marshall Farms, 5800 Lake Bluff Rd, North Rose, NY, USA), were included into the study and assigned to one of two groups, ensuring uniform distribution of sex, age, body weight (BW), and body condition score (BCS) between groups. Body condition was scored by three independent observers according to a 9-point scale [12]. Each group contained 3 females and 1 male dog with a mean age of 5.0 (SD4.2) and 5.5 (SD2.7) years, a mean body weight of 11.6 (SD1.5) and 11.6 (SD1.5) kg and a mean BCS of 6/9 (SD0.8) and 6/9 (SD0.8), for group A and B respectively. Group A contained 3 spayed females and 1 intact male, group B consisted of 3 intact females and 1 spayed male. The dogs were individually housed in large kennels with unlimited fresh water supply and free access to outdoor areas, except during the 5 days of sample collection, when metabolic cages with a wire grid floor were used in order to allow collection of all urinary and faecal output. All dogs received extra attention, cage enrichment, and controlled walks during the collection period. Prior to the study, a thorough clinical examination was performed, including blood and urine collections to ensure the dogs were in good health and habituated to all procedures and environmental stimuli.

### Experimental diets

Two experimental extruded diets were produced to contain either a soluble fibre mix of 2 % guar gum (Altaquimica, Barcelona, Spain; 200 mesh, viscosity 5000 cps after 2 to 24 h at 1 % solution at 25 °C using Brokfield DVE viscosimeter RV DVII+ set at 20 RPM Spindle No.3) and 6 % sugar beet pulp (Distribuciones Agropecuarias del Aragón, Zaragosa, Spain), hereinafter referred to as SF Diet; or 4.7 % cellulose (Arbocel BWW40, J. Rettenmaier & Söhne GMBH + CO KG, Rosenberg, Germany), hereinafter referred to as IF Diet. These fibres were supplemented in amounts to obtain similar total dietary fibre (TDF) contents for both diets (9.4 % and 8.2 % for the SF and IF diet, respectively). The analysed chemical composition of the diets is shown in Table 1. The two diets, only differing in fibre composition, were both low in protein (4 g /100 kcal ME), phosphorus, and sodium, while higher in fat, especially omega-3 fatty acids (optimal renal-protective diet according to the current state of practice) compared to regular canine maintenance diets.

**Table 1:** Composition of the diets containing fermentable fibre (SF) and non-fermentable fibre (IF) on DM basis

	SF Diet	IF Diet
OM (%)	94.9	95.2
Crude Protein (%)	16.6	16.7
Crude Fat (%)	14.6	13.5
Crude Fibre (%)	1.8	1.8
TDF (%)	10.1	9.0
Ash (%)	5.1	4.8
NFE^a^ (%)	61.8	63.2
ME^b^ (MJ/100 g)	1.755	1.737

IF, insoluble fibre; SF, soluble fibre; DM, dry matter; OM, organic matter (calculated as 100 % - ash%); TDF, total dietary fibre; NFE, nitrogen-free extract; ME, metabolizable energy

^a^NFE (% DM) was calculated as 100 - crude protein - crude fat - crude ash - crude fibre, with all components on DM basis

^b^ME was calculated as ((((5.7 x g protein) + (9.4 x g fat) + (4.1 x (g NFE + g fibre))) x (91.2 - (1.43 x % CF in DM)) /100) - (1.04 x g protein))/1000 [14]

The diets were fed in amounts sufficient to meet the daily maintenance requirement of each dog [13] (based on animal’s ideal body weight) and to maintain animal’s body weight constant throughout the study. The dogs were fed once daily, in the morning, and their body weight was recorded twice a week.

### Sample collection

On the last day of each adaptation period, blood samples (13 mL per dog) were collected via jugular venipuncture following an overnight fast and immediately distributed into vacutainers containing lithium heparin (for biochemical analysis) or tripotassium EDTA (for deuterium analysis). For pre- and postprandial acylcarnitine assay, on the last day of each collection period, blood was drawn from an aseptically placed cephalic catheter ensuring that a small amount of blood and saline flush from earlier collection were discarded before a fresh sample (3 mL, distributed into a vacutainer containing lithium heparin) was aspirated and this was done at 30 min preprandially, 1 and 2 hr postprandially, and then, every 30 min for 4 more hours. Samples were centrifuged at 1500 g for 10 min at 4 °C. Supernatants were collected, aliquoted and frozen at −20 °C awaiting further analysis.

Additionally, total faecal collection was performed over a 5-day period, while on the last day, fresh faecal samples were collected manually during a routine rectal and anal gland examination. All faecal samples were frozen upon collection. Total urine output was quantified over this 5-day collection period, and a fresh 20 mL sample was collected daily and immediately frozen until further analysis.

### Chemical analyses

The experimental diets were subjected to proximate analysis (Weende). They were dried to a constant weight at 103 °C to determine dry matter (DM, ISO 1442, 1997), while crude ash was determined by combustion at 550 °C (ISO 936, 1998). Crude protein was calculated from Kjeldahl nitrogen (6.25 x N, ISO 5983–1, 2005). Crude fibre was analysed by acid-alkali digestion (ISO 5498, 1981), and crude fat was analysed using acid-hydrolysis followed by Soxhlet extraction (ISO 1443, 1973). Nitrogen free extract (NFE) was calculated by subtracting crude ash, crude protein, crude fat, and crude fibre on DM basis from 100. Total dietary fibre (TDF) was analysed as described by Prosky et al. [14]. Fresh faecal consistency (1: very hard and dry; 2: firm, but not hard; 3: log-like; 4: very moist; 5: very moist but has distinct shape; 6: has texture, but no defined shape; 7: watery, no texture, flat) was evaluated using Purina® Faecal Scoring System [15]. Fresh faecal pH was measured in triplicates using a portable pH meter (Hanna Instruments, Temse, Belgium).

Faecal samples were lyophilized, sieved through a 1 mm mesh for hair removal, grounded up in a grinding mill (1 mm mesh, Brabender Rotary Mill; Brabender GmbH & Co. KG, Duisburg, Germany), and analysed via proximate analyses as described above for the experimental diets. These processed faecal samples were also analysed for faecal bacterial nitrogen content by the method of Mason [16], with adaptations previously described by Hesta et al. [17].

Additionally, short-chain fatty acids (SCFAs) and ammonia (NH_3_) concentrations were analysed by initially adding 5 mL of 2 % formic acid containing an internal standard (10 mg 2-ethyl butyric acid/mL formic acid) to 1 g of faeces. After 15 min centrifugation at 4 °C and 22,000 g, the supernatant was filtered and an aliquot was transferred into a 1.5 mL glass vial. Samples were stored at 4 °C until SCFA analysis using gas chromatography on a Shimadzu 2010 apparatus (Shimadzu Corporation,‘s-Hertogenbosch, The Netherlands) equipped with a Nukol column (30 m × 0.25 mm × 0.25 μm, Supelco) and a flame ionization detector. Briefly, 0.5 μl of sample was injected with the carrier gas N2, the injector temperature was 250 °C and the inlet pressure 52.7 kPa. The temperature program was 90 °C at the start of the injection, increasing 20 °C/min until 160 °C (kept for 8.5 min), furthermore increasing 10 °C/min until 170 °C (kept for 2 min). The detection temperature was 250 °C [18]. Ammonia was analysed based on a catalysed indophenol reaction and spectrophotometric analysis as described by Chaney and Marbach [19].

Plasma acylcarnitine (free carnitine, acetyl-, propionyl-, butyryl- + isobutyryl-, isovaleryl- + 2-methylbutyryl-, 3-hydroxy(OH)-isovaleryl-, 3-OH-butyryl-, tiglyl- + 3-methylcrotonyl-, methylmalonyl- and 3-OH-3-methylglutarylcarnitine) and amino acid profiles (valine, leucine, methionine, phenylalanine, tyrosine, glycine, alanine, ornithine, and citrulline) were analysed using LC-MS/MS [20], and area under the curve (AUC) was calculated using trapezoidal approximation [21].

### Deuterium analysis

Total body water composition was estimated using a stable isotope of deuterium oxide (D_2_0), a method validated in beagles [22]. A 1.11 g/mL deuterium oxide (D_2_0) solution was prepared by mixing 4.5 g NaCl (SA Fagron NV, Waregem, Belgium) with 500 mL 99.9 % D_2_0 (Euriso-Top SA, Saint-Aubin Cedex, France). After autoclaving procedure to obtain a sterile solution, the dogs received a subcutaneous injection of 0.5 g D_2_0/kg for body composition determination. Fasting blood samples (5 mL) were collected after 3 h of water deprivation, at the time of D_2_0 injection (T0), and for 4 h thereafter (T4). Plasma samples were frozen at −20 °C in sealed vials until analysis for D_2_0 enrichment by FTIR spectrometry [22]. Total body water (TBW) composition was determined as follows:

$$ \mathrm{T}\mathrm{B}\mathrm{W}\ \left(\mathrm{kg}\right)=\frac{\mathrm{injected}\ \left[{\mathrm{D}}_20\right]\ \left(\mathrm{g}\right)\ *\ 0.999\ *\ 1000}{\mathrm{T}4\left[{\mathrm{D}}_20\right]\ \left(\mathrm{ppm}\right)-\mathrm{T}0\ \left[{\mathrm{D}}_20\right]\ \left(\mathrm{ppm}\right),\ \mathrm{enrichment}\ {\mathrm{D}}_20\ \mathrm{in}\ \mathrm{plasma}} $$

and corrected by subtracting body weight (kg)*0.041, a non-aqueous exchange factor (TBW corr) [23], and shown as percent of body weight (%TBW). Hydration of fat-free mass (FFM) in dogs was assumed to be 74.4 %, thus FFM (kg) = TBW (kg) / 0.744 [24]. Fat mass (FM) was calculated by the difference between body weight and fat-free mass (FFM), and expressed as percent of body weight:

$$ \mathrm{F}\mathrm{M}\ \left(\%\right) = \frac{\mathrm{body}\ \mathrm{weight}\ \left(\mathrm{kg}\right)\hbox{--}\ \mathrm{F}\mathrm{F}\mathrm{M}\ \left(\mathrm{kg}\right)}{\mathrm{body}\ \mathrm{weight}\ \left(\mathrm{kg}\right)}*100\% $$

### Digestibility and nitrogen balance calculations

Mean apparent nutrient digestibility coefficients were calculated according to Cullison [25]:

$$ \mathrm{Nutrient}\ \mathrm{digestibility}\ \left(\%\right) = \frac{\mathrm{Nutrient}\ \mathrm{intake}-\mathrm{Nutrient}\ \mathrm{excretion}}{\mathrm{Nutrient}\ \mathrm{intake}}*100\% $$

Nitrogen balance was calculated on DM basis as g N/day.

$$ \mathrm{N}\mathrm{B}=\mathrm{N}\ \mathrm{intake}-\mathrm{faecal}\ \mathrm{N}-\mathrm{urinary}\ \mathrm{N} $$

### Statistical analysis

For all data, normality of distribution was confirmed using the Kolmogorov-Smirnov and Shapiro-Wilk tests and equality of variance was checked with Levene’s test, prior to further analyses. All data from the study were analysed in a mixed model with diet and group as fixed effects and with dog subject randomized within group. For all analyses, Superior Performing Software Systems version 19 (SPSS Inc., Chicago, Illinois, USA) was used. Statistical significance was set at *P* < 0.05 and a *P* value between 0.1 and 0.05 was defined as a trend.

## Results

### Energy intake, body weight and composition

Both test diets were well tolerated and none of the dogs showed signs of illness or maldigestion. Daily energy intakes did not differ between diets (*P* = 0.65) and body weight remained stable for all the dogs during the entire study (*P* = 0.84). There were no significant differences in fat mass and total body water percentage with respect to the different test diets (*P* = 0.99 and *P* = 0.99, respectively).

### Faecal parameters

Faecal parameters are shown in Table 2. Dogs on the SF diet produced significantly more faeces than those on the IF diet (*P* = 0.02). The faecal pH was lower in SF-fed dogs compared to the IF-fed dogs (*P* < 0.01), as was the faecal DM% (*P* < 0.01). Dogs on the SF diet showed significantly higher faecal excretions of acetic and propionic acid (*P* < 0.01 and *P* < 0.01, respectively), resulting in a higher total SCFA excretion (*P* = 0.02) compared to dogs on the IF diet. No significant diet effect was seen for faecal consistency score or faecal excretions of butyric-, isobutyric-, isovaleric- and valeric acid and NH_3_.

**Table 2:** Faecal characteristics and SCFA and NH_3_ excretion in dogs fed the SF and IF diet

	SF Diet		IF Diet		*P*-value	
	Mean	SD	Mean	SD	Group	Diet
Total faecal production (g FM/day)	81	8	61	17	NS	0.02
Faecal consistency (1–7)^a^	2	0.5	2	0.2	NS	NS
Faecal pH	5.7	0.2	6.5	0.6	NS	<0.01
Faecal DM (%)	32.8	1.8	43.2	4.5	NS	<0.01
Acetic acid (mmol/day)	8.12	1.07	4.99	1.94	NS	<0.01
Propionic acid (mmol/day)	4.90	1.00	2.58	1.11	NS	<0.01
Butyric acid (mmol/day)	1.14	0.62	0.90	0.47	NS	NS
Isobutyric acid (mmol/day)	0.10	0.02	0.10	0.05	0.03	NS
Isovaleric acid (mmol/day)	0.35	0.13	0.35	0.16	NS	NS
Valeric acid (mmol/day)	0.03	0.03	0.03	0.01	NS	NS
Total SCFA (mmol/day)^b^	14.6	2.0	8.96	3.5	NS	0.02
Faecal NH_3_ (mg/day)	30.7	9.0	31.8	9.0	NS	NS

FM, fresh matter; NS, not significant

^a^Purina faecal scoring system (1: very hard and dry; 2: firm, but not hard; 3: log-like; 4: very moist; 5: very moist but has distinct shape; 6: has texture, but no defined shape; 7: watery, no texture, flat)

^b^Total SCFA = acetic + propionic + butyric + isobutyric + isovaleric + valeric acids

### Apparent protein digestibility coefficients & N-balance

There was a significantly lower crude protein digestibility of the SF diet (*P* = 0.02). The mean apparent protein digestibility coefficients of SF and IF diets were 76.5 % (SD3.3) and 80.6 % (SD3.3), respectively. The digestibility of dry matter, organic matter, fat and NFE was not significantly different between diets (data not shown).

Dogs on the SF-diet had a slightly, but significantly lowered nitrogen intake (*P* < 0.01), however, dietary digestible protein intake was not affected (*P* = 0.68). Nitrogen balance was slightly positive but unaffected by diet (*P* = 0.35). However, total daily faecal N excretion tended to be higher with SF (*P* = 0.05), but urinary N excretion was not (*P* = 0.24). As expected, daily bacterial N excretion and faecal bacterial excretion per g of dietary N intake (data not shown) were significantly higher with SF (*P* = 0.03 and 0.04 respectively) (Table 3).

**Table 3:** Nitrogen balance in dogs fed the SF and IF diet

	SF Diet		IF Diet		*P*-value	
	Mean	SD	Mean	SD	Group	Diet
Digestible protein intake (g/day)	22.2	3.5	22.4	2.9	NS	NS
Nitrogen intake^a^ (g/day)	4.4	0.6	4.3	0.6	NS	<0.01
Total faecal nitrogen (g/day)	1.0	0.1	0.8	0.2	NS	0.05
Bacterial nitrogen (g/day)	0.20	0.04	0.16	0.03	NS	0.03
Urine nitrogen (g/day)	2.5	0.9	3.0	1.0	NS	NS
Nitrogen balance (g/day)	0.8	0.8	0.4	0.9	NS	NS

^a^Nitrogen intake was calculated as diet crude protein intake / 6.25

### Fermentation end product metabolites in plasma and amino acid profiles

Plasma acylcarnitine profile was used as a marker for fatty acid oxidation in the mitochondria, as acylcarnitine concentrations are representative of the mitochondrial acyl-CoA pool, while AA (glycine, alanine, valine, leucine, methionine, phenylalanine and tyrosine) were measured to evaluate sparing in favor of other substrates of the TCA cycle [26].

Regarding the postprandial data for the first three hours after the meal, a group effect was found for leucine + isoleucine, methionine, phenylalanine and tyrosine’s AUC (*P* < 0.01, and *P* = 0.02, 0.04, 0.01, respectively), as well as a trend for 3-OH-isovaleryl’s AUC (*P* = 0.05). No treatment effects were found for free-, acetyl-, propionyl- and isovaleryl- carnitine’s AUC, nor for glycine, valine, ornithine, methionine and citrulline’s AUC. However, compared to cellulose, SF resulted in a significant rise in butyryl- + isobtyryl-, 3-OH-butyryl-, 3-OH-isovalerylcarnitine’s AUC (*P* < 0.01 and *P* = 0.03, 0.03, respectively), as well as plasma leucine + isoleucine, phenylalanine and tyrosine’s AUC (*P* < 0.01 and *P* = 0.02, 0.04, respectively). A trend towards increased malonyl-L-carnitine’s AUC (*P* = 0.09) was also found for the SF diet. Furthermore, the citrulline/ornithine ratio was lower (*P* < 0.01) in SF-fed dogs (Table 4).

**Table 4:** Area under the curves (AUC) for the first three post-prandial hours (nmol/mL per 195 min)

		SF Diet		IF Diet		*P*-value	
Symbol	Trivial name	Mean	SD	Mean	SD	Group	Diet
Plasma acylcarnitine profile (AUC)							
C0	Free carnitine	5550.9	905.5	5730.3	857.8	NS	NS
C2	Acetyl carnitine	477.2	192.2	415.4	144.8	NS	NS
C3	Propionyl carnitine	44.9	16.3	34.9	10.9	NS	NS
C4	Butyryl + Isobutyryl carnitine	70.5	34.2	34.4	8.9	NS	<0.01
C5	Isovaleryl carnitine, 3-	40.2	9.7	33.2	7.5	NS	NS
	or 2-Methylbutyryl carnitine						
3OH-C4	3- or b-hydroxy butyryl carnitine	8.1	3.8	5.0	2.0	NS	0.03
3OH-C5	3-Hydroxy-isovaleryl carnitine	40.0	22.9	22.1	4.9	0.05	0.03
C3DC	Malonyl-L-Carnitine	4.8	3.4	2.6	0.5	NS	0.09
C4DC	Methylmalonyl or Succinyl carnitine	11.6	3.9	8.7	1.9	NS	NS
Plasma amino acid profile (AUC)							
Gly	Glycine	37061.5	6926.3	35606.5	7872.9	NS	NS
Ala	Alanine	12422.6	31817.2	100995.5	16122.9	NS	NS
Val	Valine	31261.0	3000.4	30484.3	5729.5	NS	NS
Leu	Leucine + Isoleucine	29483.5	3201.4	23726.8	5869.7	<0.01	<0.01
Orn	Ornithine	3194.6	791.7	2733.9	922.9	NS	NS
Met	Methionine	12199.7	750.4	11270.7	2351.2	0.02	NS
Phe	Phenylalanine	10036.0	887.4	7696.7	2760.1	0.04	0.02
Cit	Citrulline	10924.5	5001.4	13217.3	5684.5	NS	NS
Tyr	Tyrosine	7348.3	628.9	6137.7	1727.5	0.01	0.04
Cit/Orn	Citrulline/ornithine ratio	3.3	0.9	4.8	0.9	NS	<0.01

Regarding the postprandial data for hours three to six after the meal, a group effect was found for butyryl + isobutyryl carnitine, 3-OH-isovaleryl carnitine, malonyl-L-carnitine, methionine and tyrosine’s AUC (*P* = 0.03, 0.04, <0.01, 0.02, 0.02, respectively). Trends regarding group effect were seen for leucine + isoleucine, phenylalanine and the citrulline/ornithine ratio’s AUC (*P* = 0.07, 0.08, 0.08, respectively). A diet effect was seen for acetyl-, propionyl-, butyryl + isobutyryl-, 3-OH-butyryl-, 3-OH-isovaleryl-, malonyl-L-carnitine and leucine + isoleucine’s AUC (*P* = 0.04, 0.04, <0.01, <0.01, <0.01, 0.01, 0.01, respectively), resulting in significantly higher AUC values in the SF diet fed dogs. Isovaleryl- and methylmalonylcarnitine’s AUC, as well as phenylalanine and tyrosine’s AUC showed a similar trend (*P* = 0.06, 0.08, 0.09, 0.06, respectively). Additionally, the citrulline/ornithine’s AUC ratio was significantly lower in the three to six hour postprandial window for SF-fed dogs (*P* < 0.01). For free carnitine’s AUC, as well as for the amino acid AUC’s of glycine, alanine, valine, ornithine, methionine and citrulline, no significant diet effect was observed (Table 5). Blood urea nitrogen (BUN), total protein (TP) and albumin (ALB) were not affected by diet or group(data not shown).

**Table 5:** Area under the curves (AUC) for hours three to six postprandially (nmol/mL per 195 min)

		SF Diet		IF Diet		*P*-value	
Symbol	Trivial name	Mean	SD	Mean	SD	Group	Diet
Plasma acylcarnitine profile (AUC)							
C0	Free carnitine	6573.5	944.6	6799.1	859.3	NS	NS
C2	Acetyl carnitine	494.4	256.1	294.0	50.3	NS	0.04
C3	Propionyl carnitine	48.6	16.8	33.8	6.9	NS	0.04
C4	Butyryl + Isobutyryl carnitine	78.4	42.5	35.0	5.0	0.03	<0.01
C5	Isovaleryl carnitine, 3-	43.3	11.2	36.9	7.8	NS	0.06
	or 2-Methylbutyryl carnitine						
3OH-C4	3- or b-hydroxy butyryl carnitine	8.3	2.3	5.0	0.7	NS	<0.01
3OH-C5	3-Hydroxy-isovaleryl carnitine	42.4	18.4	22.3	3.1	0.04	<0.01
C3DC	Malonyl-L-Carnitine	5.0	3.2	2.4	0.4	<0.01	0.01
C4DC	Methylmalonyl or Succinyl carnitine	9.9	2.0	7.8	2.5	NS	0.08
Plasma amino acid profile (AUC)							
Gly	Glycine	37525.4	9943.3	38286.3	6521.0	NS	NS
Ala	Alanine	128622.7	46942.2	118073.8	31283.0	NS	NS
Val	Valine	37175.3	3055.4	36034.5	8578.4	NS	NS
Leu	Leucine + Isoleucine	34186.8	4728.2	29691.2	7663.0	0.07	0.01
Orn	Ornithine	3233.7	777.1	3045.4	1156.7	NS	NS
Met	Methionine	16235.4	1377.0	15971.2	3528.9	0.02	NS
Phe	Phenylalanine	10421.0	1665.6	8482.4	2782.3	0.08	0.09
Cit	Citrulline	11044.7	4896.4	16039.0	6558.6	NS	NS
Tyr	Tyrosine	8278.0	688.0	7170.5	1659.4	0.02	0.06
Cit/Orn	Citrulline/ornithine ratio	3.3	0.9	5.3	1.1	0.08	<0.01

## Discussion

The present study showed that SF supplementation to a low protein (renal type) diet changes the acyl carnitine profile and spares amino acids in healthy adult dogs. Moreover, faecal N excretion tended to increase, but urinary N excretion did not decrease.

As both test diets contained 33 % corn, 23 % rice and 4 % sugar, the need for gluconeogenesis in the first three hours after the meal may have been low as glucose is absorbed from the intestine [27]. After roughly 3 h [28], digesta arrive in the large intestine, where propionate can be produced and can potentially be used for gluconeogenesis. These postprandial fluctuations in gluconeogenesis seem to be reflected in the measurements of fermentation end product metabolites and amino acid profiles in this study, as there are many more dietary-induced differences, in the 3-6 h postprandial period compared to the 0-3 h postprandial period. It will however be important to consider that feeding pattern may alter these dynamics when translating these results into practice. The once a day feeding regimen that was used in this study may not translate over to twice a day feeding, which is typically preferred by pet owners.

The simultaneous rise in levels of acetyl-, propionyl-, butyryl + isobutyryl, isovaleryl-, 3-OH-butyryl-, 3-OH-isovaleryl-, malonyl-L- and methylmalonyl-CoA biosynthesis (measured in plasma by the respective carnitines) in the 3 to 6 h period in SF compared to IF diets indicate a higher supply by microbial fermentation. An increase of propionyl carnitine indicates an enhancement in production of fermentation derived propionic acid [29]. Methylmalonyl-CoA is subsequently formed from propionyl-CoA, and can be converted further into succinyl-CoA that enters into the citric acid cycle potentially releasing alternative precursors for gluconeogenesis.

In previous trials, increased production of propionic acid due to intestinal fermentation of oligofructose and inulin in domestic cats resulted in increased fasting plasma propionyl carnitine concentrations, and, contrary to the current study, a reduced fasting plasma methylmalonylcarnitine concentration (also decreased aspartate aminotransferase activity) [6]. It could be hypothesized that the plasma methylmalonylcarnitine concentrations in the current study were higher compared to Verbrugghe et al. [7] because of species differences, or because of a lower protein intake in the dogs in our study (17 % on DM) compared to cats (39 % on DM in the study by Verbrugghe et al. [7]). A negative correlation between protein intake and plasma methylmalonyl concentrations was already postulated by Rochus et al. [30], with lower protein intakes resulting in higher breakdown of valine and isoleucine therefore overriding the potential sparing of amino acids by propionic acid.

The described increase in fermentation end product metabolites was observed simultaneously with an increase in leucine + isoleucine, phenylalanine and tyrosine’s AUC in SF-fed dogs in both postprandial time periods. It could be hypothesized that increased formation of fermentation-derived acetic acid resulted in reduced conversion of leucine + isoleucine, phenylalanine and tyrosine to acetyl-CoA. Furthermore, the concerned amino acids may be related to some degree since isoleucine and leucine may be outcompeting transport of phenylalanine or tyrosine. The spared branched-chain amino acids leucine and isoleucine could in fact provide a potential effective nutritional modality for the treatment of liver disease, as opposed to sparing of the aromatic amino acids tyrosine and phenylalanine [31]. Additionally, a significant decrease in the citrulline/ornithine AUC was discovered for both post-prandial time frames. This significant decrease in the citrulline/ornithine ratio in dogs on the SF diet could be indicative of a reduced urea production and therefore, a reduction in usage of amino acids as energy source as we hypothesise the protein turnover remains unchanged. This tends to be confirmed by the increased plasma AUC of some amino acids. However, the preprandial blood urea nitrogen concentration was not reduced in the present study (a post-prandial BUN curve could have been of interest here, this was however not measured). The detected group effects found for fermentation end product metabolites and amino acid profiles could not be explained by the authors, this could possibly imply inter-animal differences.

The fact that the dogs were fed a low protein diet in this study might have contributed to the observed amino acid sparing effect as well. When feeding a low protein diet, nitrogen is in high demand for bacterial biosynthesis, and an increased blood nitrogen gradient (N-trapping) effect has been proposed with soluble fibre supplementation [10, 11]. Nitrogen trap due to increased fibre fermentation would result in a decrease in urinary nitrogen excretion, which was not observed in this study. There was, however, a trend for higher faecal nitrogen excretion for dogs on the SF diet. It could be hypothesized that the fibre fermentation was not high enough in the healthy animals to see any significant differences, a N-trap effect could possibly be more pronounced in dogs with higher blood urea concentrations and therefore, possibly a more pronounced N-flux from the blood to the gastrointestinal tract. It would be of benefit to conduct future research to evaluate this effect in dogs with renal disease.

Fortunately, despite the fact that the protein content of the diets was relatively low, the nitrogen balance remained positive. Particularly in dogs with renal or liver disease, it is important to provide an adequate amount of dietary protein in order to meet requirements to prevent malnutrition, but not to exceed these levels in order to maintain a neutral nitrogen balance [32, 33]. The nitrogen balance was comparable between the two diets, however a significant diet effect regarding the nitrogen intake was observed. However since the difference was only 0.1 gram per dog per day, this is probably irrelevant.

Faecal output was significantly increased in dogs on the SF diet, as a consequence of significantly higher faecal moisture content. This could possibly be explained by the osmotic effect of SCFA, shown in higher concentrations in faeces of dogs on the SF diet. However, even though the faecal moisture content was significantly higher, negative effects on faecal consistency score were not seen throughout the entire study, indicating a greater water-holding capacity in SF [9].

Faecal pH decreased significantly in dogs on the SF diet. This could be the consequence of thriving of intestinal lactic acid producing bacteria on this diet (daily faecal bacterial N excretion was significantly higher in dogs on the SF diet, therefore possibly indicating bacterial growth), or the effect of concurrently increased production of other SCFA in the hindgut (not measured in this study), as a result of fermentation. This could be demonstrated by the significantly higher faecal acetic and propionic acid excretion on the SF diet, resulting in a higher total SCFA excretion in dogs on the SF diet, as well as its plasma metabolites (as described above).

A significant decrease in apparent crude protein digestibility was observed with the SF diet compared to the IF diet. Several studies in dogs and cats have demonstrated that incorporation of fermentable fibre in the diet decreases apparent nitrogen digestibility [1, 4, 34, 35]. Dietary fibre can accelerate intestinal transit and have a laxative effect [36], therefore reducing time available for digestion [37]. Viscous soluble fibres can form gels in aqueous solutions, thereby binding nutrients and preventing their transfer to the absorptive surface of the intestine, possibly impairing digestion and absorption in the small intestine [38, 39]. However, these processes would be expected to affect digestion and absorption of other macronutrients (i.e. fat) as well, which was not observed. Furthermore, no differences in faecal concentrations of NH_3_, valeric or isovaleric acid were observed in dogs fed the SF and IF diets, that could have indicated an increase in protein fermentation due to a large load of undigested protein reaching the large intestine. Another possible explanation is that the soluble fibre mix might have stimulated microbial protein synthesis and turnover, as a significantly higher percentage of bacterial nitrogen was observed in the dogs fed the SF diet, resulting in a lower apparent protein digestibility. Although comparable with another study of renal diets [40], apparent crude protein digestibility of either diet may appear low for a renal diet. This can be explained by the endogenous faecal nitrogen losses, originating for instance from desquamated cells, mucoprotein and digestive enzymes, which contribute to a relatively larger part of the total faecal nitrogen excretion in low-protein diets compared to high-protein diets. In diets with identical true protein digestibility the difference between true and apparent digestibility will in fact become larger at lower dietary protein levels [41].

The experimental diets in this study were fed in amounts to meet each dog’s maintenance energy requirement at current body weight. In doing so, the animals successfully maintained their body weight throughout the entire study. Also, body composition analysis (fat mass and total body water percentage) was not different between diets, indicating that the spared amino acids were not used to increase muscular mass and no muscle loss was present to alter the nitrogen balance. Moreover, plasma urea, total protein, albumin and creatinine were not influenced by the diet. Further research is warranted to understand where the potentially spared amino acids could be used, and this in healthy dogs as well as in dogs suffering from renal or liver disease where clinical utility could be applied. A possible destination that could be explored is the immune system, as a study by Bounous and Kongshavn [42] showed that an increased dietary amino acid intake resulted in an enhanced immune responsiveness. Another potential benefit of amino acid sparing could be lean body mass development.

The described amino acid sparing effect is an important concept that could have its application in the nutritional approach to kidney or liver problems, where it would allow less protein catabolism and aid in elimination of waste products of protein metabolism (ammonia) and normalization of metabolic abnormalities (azotemia). Soluble fibre supplementation of a low protein diet could therefore lead to a reduced incidence of uremic crises and prolonged patient survival. Further research into amino acid sparing effect of fermentable fibres in dogs suffering from renal or liver disease is needed.

## Conclusion

Although there was no indication of lower amino acid deamination, these results suggest that guar gum and sugar beet pulp fermentation in the dog’s large intestine could diminish postprandial use of amino acids turning instead to the use of short-chain fatty acids as substrate for the citric acid cycle. The fact that the dogs were fed a low protein diet in this study might have contributed to the observed amino acid sparing effect of fermentable fibres. Conducting a similar study to evaluate the potential benefits of soluble fibre supplemented diets in animals with kidney or liver disease instead of healthy dogs as done herein, is of clinical interest.
